# Venetoclax‐Based Therapy for Early Relapse in Acute Myeloid Leukemia After Allogeneic Hematopoietic Stem Cell Transplantation: A Case Report and Minireview

**DOI:** 10.1002/cnr2.70450

**Published:** 2025-12-29

**Authors:** Yufeng Du, Mohammad Arian Hassani, Chunhong Li, Zhijia Zhao, Yikun Liu, Chengtao Zhang, Jinsong Yan

**Affiliations:** ^1^ Department of Hematology, Dalian Key Laboratory of Hematology, Liaoning Medical Center for Hematopoietic Stem Cell Transplantation The Second Hospital of Dalian Medical University Dalian China; ^2^ Liaoning Key Laboratory of Hematopoietic Stem Cell Transplantation and Translational Medicine Blood Stem Cell Transplantation Institute, Dalian Medical University Dalian China; ^3^ School of Public Health Dalian Medical University Dalian China; ^4^ Department of Pediatrics, Pediatric Oncology and Hematology Center Diamond Bay Institute of Hematology, The Second Hospital of Dalian Medical University Dalian China

**Keywords:** acute myeloid leukemia, allogeneic hematopoietic stem cell transplantation, azacitidine, donor lymphocyte infusion, refractory/relapsed, venetoclax

## Abstract

**Background:**

Refractory/relapsed acute myeloid leukemia (R/R‐AML) typically exhibits resistance to conventional chemotherapy, resulting in a poor overall therapeutic outcome. Salvage allogeneic hematopoietic stem cell transplantation (allo‐HSCT) is the primary treatment option in such patients. However, posttransplant relapse is still a challenge, with no established effective regimens.

**Case:**

In this case report, we present the case of a 40‐year‐old male diagnosed with R/R‐AML who underwent salvage allo‐HSCT. Unfortunately, after 4 months of follow‐up, a relapse occurred. We modified the immunosuppressive therapy and administered donor lymphocyte infusion (DLI) and decitabine but failed to obtain complete remission (CR). Subsequently, a combination of venetoclax (Ven) and azacitidine (Aza), followed by the DLI regimen, was initiated. The patient achieved CR with no measurable residual disease.

**Conclusion:**

Our data suggest that the administration of Ven in combination with Aza followed by the DLI regimen used for early post‐HSCT relapsed AML could serve as a valuable reference for treating similar patients.

## 1 Introduction

Induction therapy for acute myeloid leukemia (AML) involving anthracyclines combined with cytarabine leads to complete remission (CR) in 60%–80% of patients. However, 20%–40% of patients are refractory to this regimen, and younger patients with AML achieving CR face a relapse rate of 30%–35% within 2 years [1, 2]. Salvage allogeneic hematopoietic stem cell transplantation (allo‐HSCT) is often the sole treatment option for prolonging survival in patients with refractory/relapsed AML (R/R‐AML); nevertheless, 30%–40% of patients experience a second relapse [3, 4, 5]. Those with posttransplant relapse have a grim prognosis, with overall survival (OS) rates of < 20% at 2 years for early relapse (< 6 months) and up to 40% at 2 years for late relapse (> 2 years) [6, 7, 8].

The goal of treating such patients is to provide direct anti‐leukemic activity and enhance the immune graft‐versus‐leukemia (GVL) effect. Existing treatment options are donor lymphocyte infusion (DLI), intensive chemotherapy (IC) ± DLI, second HSCT (sHSCT), and palliative care. DLI demonstrated high remission rates in cases of mixed donor chimerism and molecular relapse, yet its efficacy in hematological relapse is limited [9, 10, 11]. Combining salvage IC with DLI can reduce tumor load, enhance response rates, and extend survival. However, most patients with early relapse struggle to tolerate IC, often leading to persistent bone marrow (BM) failure and grade 3/4 hemocytopenia, elevating the risk of infection‐related mortality [12, 13, 14]. Additionally, finding suitable donors and the high treatment‐related mortality (TRM) rates from sHSCT limit its practical application [15]. Therefore, it is imperative to explore treatment options with reduced side effects that can improve survival.

The Bcl‐2 inhibitor venetoclax (Ven) in combination with hypomethylating agents (HMAs) has been used to treat elderly or IC‐ineligible patients with AML. Its use in patients with early post‐HSCT relapsed AML is still emerging. Here, we present the case of an AML patient experiencing early hematological relapse after salvage of haploidentical HSCT. The patient did not respond to DLI combined with decitabine (Dec) therapy but subsequently achieved durable CR after receiving Ven in combination with azacitidine (Aza), followed by the DLI regimen. These results suggest that this regimen could be an effective therapeutic option for patients with early hematological post‐HSCT relapsed AML.

## 2 Case

A 40‐year‐old male presented with dizziness and fatigue lasting for 1 month and was admitted to The Second Hospital of Dalian Medical University in February 2021. His routine blood test results are as follows: white blood cell count: 1.09 × 10^9^/L; hemoglobin level: 46 g/L; and platelet count: 33 × 10^9^/L. BM morphology revealed the presence of blasts: 39%. Flow cytometry examination indicated the presence of blasts: 22.25%, which expressed CD4, CD13, CD33, CD38, CD117, and HLA‐DR and partially expressed CD34. His karyotype was 46, XY,? ins (1)(q21q31q21)[5]/46, XY[2]. Next‐generation sequencing (NGS) identified a mutation in the *ASXL1* gene (NM_015338.5): exon 12: c.1934dupG (p.Gly646fs) at a proportion of 28.3%. He was diagnosed with AML‐M2.

**FIGURE 1:**
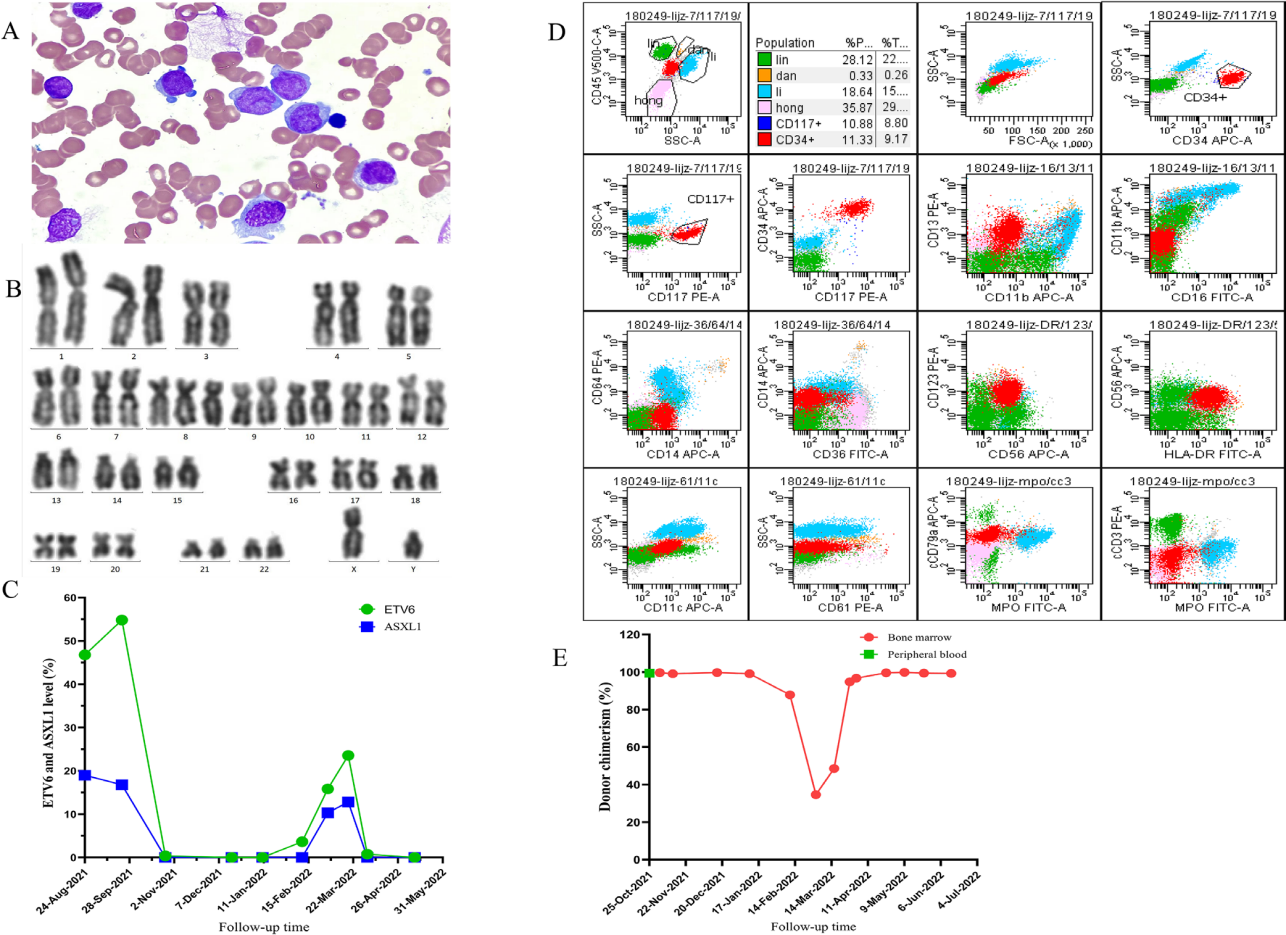
(A) Morphology of the patient's blasts (scale: 10 × 100). (B) Results of flow cytometry analysis. (C) Karyotype analysis for the patient. (D) Level changes for *ETV6* and *ASXL1*. (E) Variation of chimerism in the patient.

Initially, the patient received the DA regimen achieving CR, then the patient also received two cycles of high‐dose cytarabine (HiDAC). After completing this treatment, BM and peripheral blood examinations revealed once again the presence of increased numbers of blasts, indicating an early relapse. He was then treated with homoharringtonine, daunorubicin and cytarabine (HAD) and fludarabine, cytarabine, and granulocyte colony‐stimulating factor (FLAG) regimens, with no response. The patient was diagnosed with R/R‐AML and the disease was reevaluated from the point of view of morphology, immunology, cytogenetics, and molecular biology (Figure 1A–D). A mutation in the *ETV6* gene was identified. We decided to perform the allo‐HSCT. Given the R/R nature of the patient's AML, an IC regimen of Dec + CLAG and then the Beijing protocol, which included Bu/Cy + ATG, were administered before the conditioning regimen. Graft‐versus‐host disease (GVHD) prophylaxis regimen consisting of tacrolimus + methotrexate (tacrolimus: 0.03 mg/kg/d, starting with −1d; methotrexate: 15 mg/m^2^, d1; 10 mg/m^2^, d3, d5, d11) was also administered. Then the patient received salvage haploidentical HSCT. Later, granulocytes and megakaryocytes were engrafted. Unfortunately, after a few months of remission, the number of blasts increased again with decreased chimerism, and an increase in the proportion of *ETV6* mutation was also observed (Figure 1D,E). This was considered an early posttransplant hematological relapse (The proportion of blast cells in BM was 5%, and in peripheral blood it was 1%. The chimerism rate decreased to 87.86%.).

**FIGURE 2:**
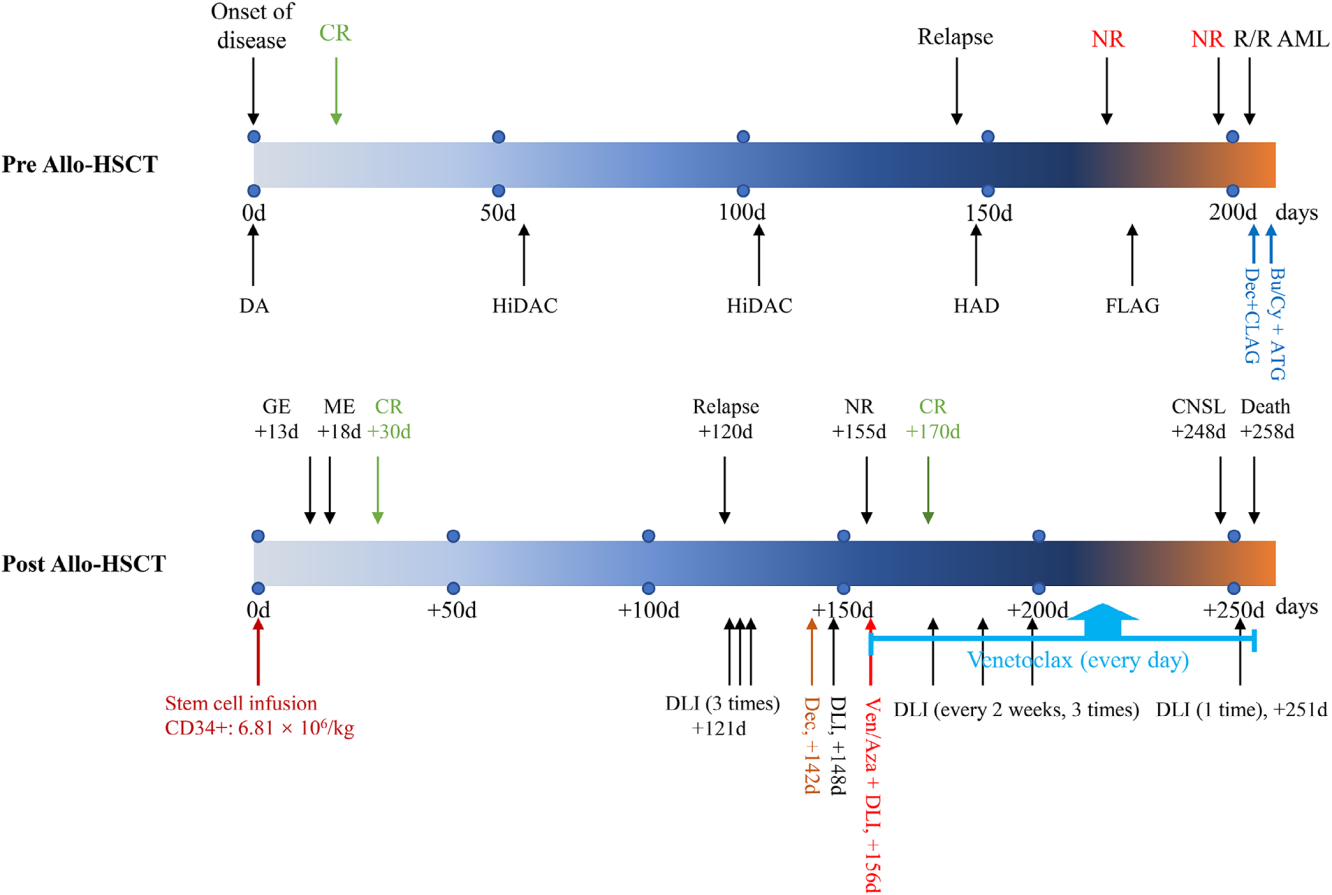
Allo‐HSCT: allogeneic hematopoietic stem cell transplantation; Bu/Cy + ATG: semustine 450 mg day −8, busulfan 62 mg Q6h days −7 to −5, cyclophosphamide 3.5 g days −4 to −3, anti‐thymoglobulin: 780 mg days −5 to −2; CNSL: central nervous system leukemia; CR: complete remission; DA: daunorubicin 140 mg days 1–3, cytarabine 200 mg Q12h days 1–7; Dec: decitabine 50 mg days 1–5; Dec + CLAG: decitabine 50 mg days 1–5, G‐CSF 300 μg days 2–6, cladribine 10 mg days 3–5, cytarabine 3.8 g days 3–6; DLI: donor lymphocyte infusion; FLAG: fludarabine 50 mg days 1–5, cytarabine 3.4 g days 1–5, G‐CSF 300 μg days 0–5; GE: granulocytes engraftment; HAD: homoharringtonine 4 mg days 1–7, daunorubicin 90 mg days 1–3, cytarabine 200 mg days 1–3; HiDAC: cytarabine 6 g Q12h days 1–3; ME: megakaryocyte engraftment; NR: no remission; R/R‐AML: relapse/refractory AML; Ven/Aza: venetoclax 200 mg [oral fluconazole] days 1–28, azacitidine 100 mg days 1–7.

The patient received DLI, Dec, and again DLI (Figure 2) but still, the therapeutic response was not favorable. We decided to administer Ven + Aza (Ven: 200 mg/day [oral fluconazole], d1–28; Aza: 75 mg/m^2^, d1–7) due to the ineffectiveness of Dec + DLI and the patient's poor fitness. At the end of the treatment, BM cytological examination showed no blasts, measurable residual disease (MRD) indicated the presence of 0.86% of blasts, and the chimerism was 94.83%, suggesting that the patient was in CR again, which persisted to the end.

During the Ven + Aza combined DLI treatment, the BM proliferation was extremely low so the hematopoiesis was promoted with granulocyte colony‐stimulating factor and danazol. We continued with DLI and Ven administration; during the treatment, the Ven dose was decreased (100 mg) due to the addition of oral posaconazole. Later, the DLI dose was also reduced. However, on day 248 after HSCT, the patient experienced a sudden onset of abnormal right‐side limb motility with dizziness and unclear speech. Cranial magnetic resonance imaging revealed abnormal foci of reinforcement in the parietal midline of the left frontal area of the brain with cerebral white matter edema, likely due to the intracerebral infiltration of leukemic cells, left lateral ventricular effusion, and subfalcine herniation. Manno fructose was used to reduce intracranial pressure. Unfortunately, 10 days later, the patient passed away with an OS of 138 days after post‐HSCT relapse. The treatment process of the patient is shown in Figure 2.

## 3 Discussion and Literature Review

Patients with R/R‐AML have a poor prognosis due to their insensitivity to several chemotherapeutic drugs. So, allo‐HSCT could be the first choice of treatment in such patients. However, a considerable number of them experience relapse within 2 years after transplantation. The mechanism of relapse after transplantation is assumed to be due to the escape of leukemic cells from the pretransplant cytotoxic killing and the immune surveillance of allogeneic T cells after transplantation [16]. Mechanisms of immune evasion may include loss of leukemia cell recognition due to loss of mismatched HLA molecules, high expression of anti‐inflammatory factors, low expression of pro‐inflammatory cytokines, release of metabolically active enzymes, and acquisition of mutations in new leukemia driver genes [17, 18].

One of the widely used treatment strategies for patients with post‐HSCT relapsed AML is to reduce immunosuppression to elevate T‐lymphocyte levels and enhance the GVL effect through DLI. DLI is an adoptive cellular immunotherapy that further augments the GVL effect observed after allo‐HSCT. The European Group for Blood and Marrow Transplantation analyzed 339 patients with post‐HSCT relapsed AML, where 171 patients received DLI and 228 did not. The results demonstrated a CR rate of 34% in the DLI group, with incidences of acute GVHD and chronic GVHD at 43% and 46%, respectively. Multivariate analysis revealed that patients with < 35% blasts at relapse and favorable cytogenetics had a better prognosis. The 2‐year OS rate was 21% ± 3% in the DLI group and 9% ± 2% in the non‐DLI group, indicating that DLI usage improves OS in patients with post‐HSCT relapse and that those with a lower tumor load tend to have a more favorable prognosis [9]. However, the higher risk of GVHD necessitates careful consideration before initiating treatment to identify the patients who are likely to benefit the most from this approach.

Another retrospective study focused on 113 patients with AML/myelodysplastic syndromes (MDS) treated with DLI given either preemptively (pDLI) or therapeutically (tDLI) posttransplantation. The results indicated that the 5‐year OS rate in the pDLI group (80%) was higher than that in the tDLI group (40%), and the cumulative incidence of GVHD was lower in the pDLI group (31%) than in the tDLI group (45%) [19]. A recent retrospective study from Japan analyzing 84 patients with AML who received DLI after haploidentical transplantation revealed a significantly higher overall response rate (ORR) and median survival time in the pDLI group (47.4%, 7.6 months) than the tDLI group (13.9%, 2.6 months) [20]. These findings suggest that DLI is more effective in patients with molecular relapse or lower tumor burden. Therefore, regular monitoring of MRD to detect molecular relapse is crucial for prolonging survival with pDLI. Table S1 presents the efficacy of DLI for acute leukemia and MDS after HSCT in the last decade [11, 21, 22, 23, 24, 25].

Patients who relapse after allo‐HSCT may achieve remission with IC if they are deemed fit to receive it. In a single‐arm multicenter study, 175 patients with AML who relapsed after transplantation attained a CR rate of 36% after one cycle of induction therapy with intermediate‐dose cytarabine ± purine analogs or anthracyclines. Among them, 42 patients received a second cycle of chemotherapy and achieved a CR rate of 25%, with a TRM of 14%. The cohort had a median survival of 6.3 months and a 2‐year OS of 18% [26]. Schmid et al. conducted a retrospective analysis of 263 patients with relapsed AML who were subsequently treated with allo‐HSCT. Of these, 47 patients were exclusively treated with an intensive induction regimen comprising medium to high doses of cytarabine and anthracyclines, resulting in a CR rate of 27% and a 2‐year OS rate of only 4.4% ± 3%. Meanwhile, 48 patients received intensive induction chemotherapy followed by DLI, achieving a CR rate of 30% and a 2‐year OS rate of 12.6% ± 5%. However, the non‐relapse mortality rate associated with IC combined with DLI was as high as 15.4% [13]. Another retrospective study compared the anti‐leukemic effects of chemotherapy alone to chemotherapy combined with DLI in 82 patients with AML who relapsed after transplantation. The group receiving chemotherapy combined with DLI had a significantly higher CR rate (64.0% vs. 12.5%, *p* < 0.01) and a significantly lower relapse rate (50.0% vs. 100.0%, *p* < 0.01) than the chemotherapy‐only group [27]. Collectively, these studies suggest that administering DLI after chemotherapy improves response rates and prolongs survival in patients with post‐HSCT relapse compared to chemotherapy alone. The possible reasons for this include the reduction of tumor burden with pre‐DLI chemotherapy and the enhanced GVL effect of DLI, which corrects lymphocyte depletion while reducing the risk of BM failure. Nevertheless, assessing patient fitness is crucial before initiating IC to minimize TRM. The application of IC ± DLI in post‐HSCT relapsed AML and MDS over the past decade is summarized in Table S2 [12, 13, 26, 27, 28, 29].

sHSCT is another therapeutic option for post‐HSCT relapse. An analysis of 418 patients with AML who experienced HSCT relapse and were subsequently treated with either sHSCT or DLI revealed no differences in 2‐year (HSCT: 26% vs. DLI: 25%) and 5‐year (HSCT: 19% vs. DLI: 15%) OS rates [15]. However, another study showed that sHSCT achieved a high CR rate (41%) but could not maintain the CR status in the long term, resulting in a 2‐year OS of only 15% ± 8% and a high non‐relapse mortality rate (42.1%) during the treatment [13]. These findings indicate that the efficacy of allo‐sHSCT is not superior to that of DLI, and it may not be the optimal choice. Also, its application is limited in patients who have relapsed after receiving HSCT for AML, often due to their poor fitness and ineligibility for myeloablative conditioning [30, 31]. The use of allo‐sHSCT for patients with post‐HSCT relapsed myeloid neoplasms in the last decade is summarized in Table S3 [13, 15, 32, 33, 34, 35, 36, 37, 38, 39, 40, 41, 42]. Since most patients with early post‐HSCT relapse are unfit for IC and allo‐sHSCT, research on the efficacy of HMAs during the posttransplant period has been conducted. In addition to their anti‐leukemic effect, HMAs enhance the activity of regulatory and cytotoxic T cells, maintaining the effects of GVL while regulating GVHD [43]. Aza has shown promise in reducing posttransplant relapse rates and has better efficacy in cases of decreased chimerism or positive MRD. However, its posttransplant maintenance therapy has not been found to improve OS [44, 45, 46, 47]. A German multicenter retrospective study of 154 patients with early posttransplant molecular and hematological relapses (124 with AML and 28 with MDS) found that 105 patients received a median of four courses of Aza and DLI. The study revealed a higher 2‐year OS rate in the molecular relapse group than in the hematological relapse group (69% ± 16% vs. 19% ± 6%, *p* < 0.01) [48]. This suggests that Aza in combination with DLI can produce favorable outcomes as preemptive therapy but is less effective in patients with hematological relapse. Moreover, the patients included in this study were from outpatient rather than inpatient settings, indicating that this regimen has a promising safety profile and may reduce patient admission rates. A recent European multicenter study also suggested the significant efficacy of Aza + DLI for molecular relapse, with higher 1‐year event‐free survival and OS rates than the Aza group alone [49]. The study by Craddock C et al. indicated that the ORR and 2‐year OS rates of Aza ± DLI in post‐HSCT relapsed AML were 29.3% and 12%, respectively. The research concluded that the efficacy of Aza ± DLI for post‐HSCT relapsed AML was determined by the time to relapse and the percentage of blasts in the BM at the time of relapse [50]. The above studies collectively suggest that Aza ± DLI has better safety but still has limited efficacy. Therefore, it seems necessary to explore new regimens for the treatment of post‐HSCT relapsed AML. The treatment of post‐HSCT relapsed myeloid neoplasms with Aza ± DLI in the last decade is shown in Table S4 [45, 46, 47, 48, 49, 50, 51, 52, 53, 54, 55, 56, 57, 58, 59, 60, 61].

Ven is a selective Bcl‐2 inhibitor with limited efficacy in treating AML as a single agent [62]. In recent years, the use of Ven in combination with HMAs for AML in elderly or unfit patients has gained attention. An international multicenter, randomized, double‐blind, and phase III clinical trial of Ven combined with Aza enrolled 431 patients with newly diagnosed AML (286 cases in the Ven + Aza group and 145 cases in the placebo + Aza group). The CR with incomplete count recovery (CRi) rates between the two groups were 66.4% and 28.3%, respectively (*p* < 0.001), and the median OS rates were 14.7 and 9.6 months, respectively (*p* < 0.001). Ven was associated with a higher probability of fever ≥ grade 3 with neutropenia than the placebo (30% vs. 10%). Nonetheless, the early mortality rates were similar in both groups [63]. These data suggest that Ven + Aza is more effective than monotherapy. The synergistic mechanism between the two in terms of Ven resistance, amino acid metabolism, and antioxidant activity has been previously demonstrated [64, 65, 66, 67]. Aldoss et al. reported the clinical efficacy of Ven combined with HMAs in 33 cases of R/R‐AML. The median follow‐up time was 6.5 months, the CR/CRi rate was 51%, and the 1‐year OS rate was 53%. Notably, 19 of the included patients had post‐HSCT relapse, with an ORR of 46.2% and a 6‐month OS rate of 42.3% after treatment [68]. Although the patient population is small, it revealed the efficacy of Ven + Aza for treating post‐HSCT relapsed AML.

Our patient had R/R‐AML with BM blasts of > 20% at the time of allo‐HSCT. As presumed, he developed an early hematological relapse 4 months after allo‐HSCT. After the withdrawal of immunosuppressive agents and treatment with three courses of DLI, we observed an increase in the BM blasts and a progressive decrease in chimerism, suggesting that DLI alone was not effective. A retrospective German study of 36 post‐HSCT relapses of AML and MDS showed the efficacy of using Dec + DLI, with an overall ORR of 25% (CR rate: 17%, partial remission rate: 8%), a median time to CR of 10 (range: 2–33) months, and a 2‐year OS rate of 11% ± 6% [69]. Therefore, we also used the combination of Dec + DLI for our patient; however, the disease still progressed in this patient. Before HSCT, the patient had already been treated with multiple chemotherapeutic agents, and due to his poor physical status and the absence of specific molecular mutations that could be sensitive to targeted therapies, finding a treatment option was difficult. However, the efficacy of the Ven + Aza regimen for post‐HSCT relapse due to the failure of Dec therapy was uncertain [70].

Ven + Aza regimen has demonstrated favorable efficacy in newly diagnosed AML [63]. DLI is a commonly used intervention for managing posttransplant AML relapse. We hypothesize that the combination of Ven + Aza with DLI may hold potential therapeutic benefit for this patient. The patient developed acute GVHD, manifesting as acute skin rejection, following the administration of this treatment regimen, and was subsequently managed with ruxolitinib. Notably, no acute GVHD was observed when Dec + DLI was used. Whether the occurrence of acute GVHD was related to the addition of Ven was not clearly understood and needs to be verified by the inclusion of more case studies. After 2 weeks of induction, the patient reached CRi again with complete chimerism. We hypothesize that the mechanism of achieving CRi is related to the synergistic reduction of tumor burden by Ven + Aza and the enhanced GVL effect of DLI due to the regulation of T‐lymphocyte activity. The patient experienced grade 3–4 febrile neutropenia and thrombocytopenia during the treatment, which are common side effects of Ven combination regimens; the prophylactic administration of broad‐spectrum antibiotics was also necessary [71, 72]. Finally, Table S5 summarizes the efficacy of Ven‐based therapy for AML and MDS with post‐HSCT relapse [70, 71, 73, 74, 75, 76, 77, 78].

## 4 Conclusion

In summary, this case report suggests that Ven + Aza followed by DLI could be a potential treatment option for relapsed AML after HSCT and it provides a reference for future clinical studies.

## Author Contributions

**Yufeng Du:** data collection, literature search, and writing. **Mohammad Arian Hassani:** data analysis and language improvement. **Chunhong Li:** data analysis and image production. **Zhijia Zhao:** table making. **Yikun Liu:** data collection and checking. **Chengtao Zhang:** study execution, data acquisition, and revision. **Jinsong Yan:** research project design and agreement to be accountable for all aspects of the work.

## Funding

This research was supported by Central Guidance on Local Science and Technology Development Fund of Liaoning Province (No. 2023JH6/100100019).

## Ethics Statement

This study received written informed consent from patients and was approved by the ethics committee of the second affiliated hospital of Dalian Medical University.

## Conflicts of Interest

The authors declare no conflicts of interest.

## Supporting information


**Table S1:** The studies of DLI alone for the therapy of relapse after posttransplant with AL and MDS (the data were from PubMed between 2012 and 2022).

**Table S2:** The studies investigating intensive chemotherapy ± DLI as a treatment of posttransplantation relapse with AML and MDS (the data were from PubMed between 2012 and 2022).

**Table S3:** The studies of secondary hematopoietic stem cell transplantation in posttransplant relapse (the data were from PubMed between 2012 and 2022).

**Table S4:** The studies investigating AZA ± DLI as a treatment of posttransplantation (the data were from PubMed between 2012 and 2022).

**Table S5:** The studies of venetoclax‐based ± DLI therapy in AML and MDS relapse after transplantation (the data were from PubMed between 2012 and 2022).

## Data Availability

The data that support the findings of this study are available from the corresponding author upon reasonable request.
