# Supplementary material for: Venetoclax‐Based Therapy for Early Relapse in Acute Myeloid Leukemia After Allogeneic Hematopoietic Stem Cell Transplantation: A Case Report and Minireview
Source: Cancer Rep (Hoboken). 2025 Dec 29;9(1):e70450. doi: 10.1002/cnr2.70450 (PMC12747801; doi:10.1002/cnr2.70450)
Supplement: Supplementary file 1 — Table S1: The studies of DLI alone for the therapy of relapse after posttransplant with AL and MDS (the data were from PubMed between 2012 and 2022). [file CNR2-9-e70450-s003.docx]

| study | Year | Diagnose  (numbers) | Type of study | Patients  (numbers) | Median age  (years) | Type of relapse | Median time to relapse  (months) | Chemotherapy before DLI  (numbers) | DLI | | CR  (%) | ORR  (%) | Median Survival  (months) | 2-years OS  (%) | GVHD  (%) | NRM  (%) |
| --- | --- | --- | --- | --- | --- | --- | --- | --- | --- | --- | --- | --- | --- | --- | --- | --- |
|  |  |  |  |  |  |  |  |  | Median  cycles | Total median CD3+ cell numbers/patient |  |  |  |  |  |  |
| Eefting et al.  (21) | 2014 | AML (41)  MDS (3) | Retro | 44 | 53 (18–68) | Morph | 6.4 (2.2-37.2) | Not combined | NM | 6.0 (1.0-100.0) ×10^6^/kg | 33 | 33 | NM | 23 | aGVHD: 63  cGVHD: NM | 33 |
| Takami et al.  (22) | 2014 | AML (143) | Retro | 143 | 49 (16–67) | Morph | 5.0 (0.9-71.8) | 55 | ≥1 | NM | 8 | NM | NM | 17±3 | aGVHD: 18  cGVHD: NM | 29 |
| Eefting et al.  (23) | 2016 | AML (70)  MDS (9) | Retro | 79 | 55 (21-72) | Molec | 7.2 (3.0-27.3) | Not combined | 2 (1-4) | NM | NM | NM | NM | 58 | aGVHD: NM  cGVHD:NM | NM |
| Patriarca et al.  (11) | 2020 | AML (180)  ALL (72) | Retro | 252 | 45 (2–73) | Morph  Molec | NM | 40 | ≥1 | (0.01–50.0×10^6^/kg) | NM | NM | 15.5 (0.1-108.5) | 39 | aGVHD: NM  cGVHD: NM | 16 |
| Rettig et al.  (24) | 2021 | AML (51) | Retro | 51 | 56 (22-78) | Morph | 5.1 | Not combined | 1: 18%  2: 22%  ≥3: 61% | First cycle:  0.84 (0.22–3.56) ×10^6^/kg  Second cycle:  1.08 (0.31–4.59) ×10^6^/kg  Third or later cycle:  1.8 (0.34–10.0) ×10^6^/kg | NM | NM | 10.4 | 26 | aGVHD: 18  cGVHD: 10 | 4 |
| Zuanelli Brambilla et al.  (25) | 2021 | AML (7)  MDS (10) | Retro | 17 | 65 (52-71) | Morph | <6 months: 17.6%  6-12 months: 58.8%  >12 months: 23.5% | Combined HMA | 1 (1-4) | NM | NM | NM | NM | NM | aGVHD: NM  cGVHD: NM | NM |

TABLE S1. The studies of DLI alone for the therapy of relapse after post-transplant with AL and MDS (The data were from PubMed between 2012 and 2022).

AL, acute leukemia; AML, acute myeloid leukemia; ALL, acute lymphoblastic leukemia; MDS; myelodysplastic syndromes; Retro, retrospective study; Morph, morphological; Molec, molecular; HMA, hypomethylating agents; DLI, donor lymphocytes infusions; CR, complete remission; ORR, overall response rate; OS, overall survival; GvHD, graft-versus-host disease; NRM, non-relapse mortality; NM, not mentioned.
