# Supplementary material for: Venetoclax‐Based Therapy for Early Relapse in Acute Myeloid Leukemia After Allogeneic Hematopoietic Stem Cell Transplantation: A Case Report and Minireview
Source: Cancer Rep (Hoboken). 2025 Dec 29;9(1):e70450. doi: 10.1002/cnr2.70450 (PMC12747801; doi:10.1002/cnr2.70450)
Supplement: Supplementary file 2 — Table S2: The studies investigating intensive chemotherapy ± DLI as a treatment of posttransplantation relapse with AML and MDS (the data were from PubMed between 2012 and 2022). [file CNR2-9-e70450-s002.docx]

| study | Year | Type of study | diagnose | Patients  (numbers) | Median age | Type of relapse | Median time to relapse  (months) | Chemotherapy  regimens | DLI | | | CR  (%) | ORR  (%) | Median Survival  (months) | 2-years  OS  (%) | GVHD  (%) | TRM  (%) |
| --- | --- | --- | --- | --- | --- | --- | --- | --- | --- | --- | --- | --- | --- | --- | --- | --- | --- |
|  |  |  |  |  |  |  |  |  | NO. | Median  cycles | Median CD3+ cell numbers |  |  |  |  |  |  |
| Schmid  et al.  (13) | 2012 | Retro | AML (95) | Chemo:  47  Chemo + DLI:  48 | NM | Morph | 5.54  (1.0-83.0) | HDAC, Anthracyclines | 48 | NM | NM | Chemo:  27  Chemo + DLI:  30 | NM | NM | Chemo:  4.4±3  Chemo + DLI:  12.6±5 | NM | Chemo:  8.7%  Chemo + DLI:  15.4% |
| Yan  et al.  (27) | 2013 | Retro | AML (45)  ALL (37) | Chemo:  32  Chemo + DLI:  50 | Chemo:  25 (9–55)  Chemo + DLI:  22 (6-57) | Morph | NM | AA, FLAG, HAA,  MTX, CODP | 50 | NM | 44 (11–207) ×10^6^/kg | Chemo:  12.5  Chemo + DLI:  64 | NM | Chemo:  2.5(1.31-3.69)  Chemo + DLI:  5.57 (3.72–7.42) | NM | Chemo:  aGVHD 40.6  cGVHD 3.1  Chemo + DLI:  aGVHD 66.0  cGVHD 44.3 | Chemo:  0  Chemo + DLI:  14 |
| Motabi  et al.  (12) | 2016 | Retro | AML (83)  MDS (17) | Chemo:  73  HMAs:  27 | Chemo:  52 (18–70)  HMAs:  59 (25-69) | NM | Chemo:  4.9  HMAs:  3.8 | FLAG | Chemo + DLI:  41  HMAs + DLI:  9 | 1 (1-3) | Sibling:50×10^6^/kg  Unrelated:10×10^6^/kg | Chemo: 40  Chemo + DLI: 59  HMAs: 7  HMAs + DLI: 22 | Chemo: 51  Chemo + DLI: 68  HMAs: 19  HMAs + DLI: 33 | Chemo: 2.1  Chemo + DLI:  9.8  HMAs: 4.2  HMAs + DLI:  3.9 | NM | Chemo + DLI:  aGVHD 64  HMAs + DLI:  aGVHD 22 | Chemo: 8  HMAs: 7 |
| Kurnaz  et al.  (28) | 2016 | Retro | AML (26)  ALL (28) | 54 | 26 (14-57) | Morph  Molec | NM | FLAG | 54 | 2 (1-3) | 72 (27–160) ×10^6^/kg | NM | NM | NM | NM | aGVHD 18.5 | NM |
| Sun  et al.  (29) | 2019 | Retro | AML (89) | 89 | 22 (3-57) | Morph | 9.0  (1.3-62.6) | NM | 89 | NM | 40 (11–207) × 10^6^/kg | 62.9 | NM | NM | 32.2  (20.9-43.5) | aGVHD 47.6  cGVHD 46.0 | 10.3 |
| Krakow  et al.  (26) | 2022 | Retr | AML (175) | 175 | NM | Morph  Molec | NM | HDAC,  HDAC + PA,  non HDAC + PA | Not combined | | | 36 | NM | 6.3 (5.1-8.3) | 18  (13-25) | aGVHD 20.0  cGVHD 7.1 | 14 |

TABLE S2. The studies investigating intensive chemotherapy ± DLI as a treatment of post-transplantation relapse with AML and MDS (The data were from PubMed between 2012 and 2022).

AML, acute myeloid leukemia; ALL, acute lymphoblastic leukemia; MDS, myelodysplastic syndromes; CMML, chronic myelomonocytic leukemia; PMF, primary myelofibrosis; Retr, Retrospective study; Morph, morphological; Molec, molecular; chemo, Chemotherapy; AA, aclacinomycin and cytosine arabinoside; FLAG, fludarabine, cytosine arabinoside, and G-CSF; HAA, harringtonine, aclacinomycin, and cytosine arabinoside; CODP, cyclophosphamide, vincristine, daunorubicin, and prednisone; MTX, methotrexate; HDAC, high-dose cytarabine; PA, purine analogue; HMAs, hypomethylating agents; DLI, donor lymphocytes infusions; GvHD, graft-versus-host disease; TRM, Treatment related mortality. NM，not mentioned.
