# Supplementary material for: Venetoclax‐Based Therapy for Early Relapse in Acute Myeloid Leukemia After Allogeneic Hematopoietic Stem Cell Transplantation: A Case Report and Minireview
Source: Cancer Rep (Hoboken). 2025 Dec 29;9(1):e70450. doi: 10.1002/cnr2.70450 (PMC12747801; doi:10.1002/cnr2.70450)
Supplement: Supplementary file 3 — Table S3: The studies of secondary hematopoietic stem cell transplantation in posttransplant relapse (the data were from PubMed between 2012 and 2022). [file CNR2-9-e70450-s004.docx]

TABLE S3. The studies of Secondary hematopoietic stem cell transplantation in post-transplant relapse (The data were from PubMed between 2012 and 2022).

| study | Year | Type of study | diagnose | Patients  (numbers) | Median age | Median time to relapse  (months) | Donor/cell source for HSCT2 | conditioning  regimens | Median CD34+ cell numbers/kg | CR  (%) | ORR  (%) | Median Survival  (months) | OS  (%) | GVHD  (%) | TRM  (%) | NRM  (%) |  |
| --- | --- | --- | --- | --- | --- | --- | --- | --- | --- | --- | --- | --- | --- | --- | --- | --- | --- |
| Schmid et al.  (13) | | 2012 | Retro | AML | 20 | NM | 5.54 (1.0-83.0) | NM | NM | NM | 41 | NM | NM | 2-years: 15±8 | NM | NM | 42 |
| Kharfan-Dabaja et al.  (15) | | 2018 | Retro | AML | 137 | 43 (32-52) | 11.6(4.5-23.7) | BM:4.4%  PBSC:94.9%  BM+PBSC:0.7% | MAC: 35.1%  RIC: 64.9% | NM | 38.7 | NM | NM | 2-years: 26 | aGVHD:37 (28-45)  cGVHD:31 (22-39) | NM | 26(19-34) |
| Leung et al.  (32) | | 2013 | Retro | ALL: 37  AML:71 | 108 | 37 (16–57) | 7.9 (1.3-132) | RIC-PBST | ICE, FLAG, CLARA | 5.0 (0.88-14.2)×10^6^ | 67 | NM | 11.6 | 2-years: 26 | 45 | 16.7 | NM |
| Konuma et al.  (33) | | 2016 | Retro | AML: 23  ALL: 7  CML: 2  MDS: 2 | 34 | 40.5 (17-62) | 11.8 (0.4-140.8) | CB | TBI: 28  BU: 1  FLAG: 5 | 0.86 (0.21-3.08)×10^5^ | 53 | NM | NM | 3-years: 29 | aGVHD: 70  cGVHD: 67 | 27.2 | NM |
| Orti et al.  (34) | | 2016 | Retro | AML: 88  MDS: 25  MPN: 3 | 116 | 38 (4-69) | 8.1 (1.2-119.6) | PBST: 86%  CB: 9%  BMT: 5% | Flu/Bu: 30  Flu/Mel: 9  Bu/Cy: 8  TBI: 18  Others: 51 | NM | NM | NM | NM | 5-years: 32 | aGVHD: 47  cGVHD: 31.9 | NM | 32  (23.4-40.9) |
| Christopeit et al.  (35) | | 2018 | Retro | AML: 537 | 537 | 45 (18-78) | 10.0 (0.6-176.0) | BMT: 88%  CB: 12% | MAC: 46%  RIC: 54% | NM | NM | NM | 52.3 (1-167) | 3-years:39 | aGVHD: 39  cGVHD: 31 | NM | 33.7  (29.6-37.9) |
| Lim ABM et al.  (36) | | 2018 | Retro | AML: 54 | 55 | 46 (17-71) | 5.5 (0.2-123.1) | NM | NM | NM | 30 | NM | NM | 5-years: 23 | NM | NM | NM |
| Ortí et al.  (37) | | 2018 | Retro | AML: 20  ALL: 7 | 27 | 28 (4-54) | 12.6 (2.0-50.3) | PBST: 59%  other: 41% | Cy/TBI: 40%  Flu/Bu: 24%  Flu/Mel: 12%  Bu/Cy: 4%  Other: 20% | NM | NM | NM | NM | 2-years: 60 | aGVHD: 37  cGVHD: 37 | NM | 16 |
| Schneidawind et al.  (38) | | 2018 | Retro | AML: 29  ALL: 11 | 40 | 41 (18-65) | NM | PBST | Flu/Thio/Mel:43%  Clo/Thio/Mel:13%  FLAMSA-RIC:10%  Bu/Cy: 8%  TBI/Cy: 5%  Others: 23% | 7.46 (2.4-16.6)×10^6^ | NM | NM | NM | 2-years: 31 | aGVHD: 40  cGVHD: 38 | NM | 31 |
| Shimoni A et al.  (39) | | 2019 | Retro | AML: 556 | 556 | 46 (20-73) | SD: 10.6 (1-129.9)  DMD:12.5(0.6-236.2)  Haplo: 9.3(1.1-80.8) | Sib: 36%  UD: 64% | MAC: 41%  RIC: 59% | NM | NM | NM | NM | SD: 37  DMD: 29  Haplo: 23 | SD: aGVHD 35.9, cGVHD 35.9  DMD: aGVHD 32.7,  cGVHD 31  Haplo: aGVHD 20.1,  cGVHD 25.1 | NM | SD: 25.1  DMD: 26.9  Haplo: 33.9 |
| Choi et al.  (40) | | 2021 | Retro | AML: 54  ALL: 17 | 71 | 38 (17-70) | 9.0 (0.7–222.7) | PBST: 98.8%  BMT: 1.2% | Bu/Cy  Flu/Bu | 6.3 (0.2–38.3)×10^6^ | NM | NM | 10.3 | 2-years: 21  5-years: 11.8 | aGVHD: 35  cGVHD: 34 | NM | 18.7 |
| Han et al.  (41) | | 2021 | Retro | AML: 21  ALL: 7 | 28 | 28 (3–48) | 9.1 (2–62.2) | MSD: 35.7%  Haplo-donor:60.7%  URD: 3.6% | TBI based: 71%  Bu based: 29% | NM | 92.9 | 92.9 | NM | 1-years: 25  2-years: 17.9 | aGVHD: 25  cGVHD: 39 | NM | 25 |
| Kharfan-Dabaja et al.  (42) | | 2021 | Retro | AML: 455 | De-novo:  388  Secondary:  67 | MUD:  46 (35–58)  Haplo:  44 (33–53) | MUD:  15 (1-149)  Haplo:  11 (1-89) | MUD: 70%  Haplo: 30% | NM | NM | NM | NM | MUD:  10 (8-13)  Haplo:  11 (7-16) | 2-years  MUD:31  Haplo:  29 | aGVHD  MUD:30  Haplo-donor: 27  cGVHD  MUD:32  Haplo: 22 | NM | MUD: 26  Haplo: 27 |

AML, acute myeloid leukemia; ALL, acute lymphoblastic leukemia; CML, chronic myelogenous leukemia; MDS, myelodysplastic syndromes; MPN, myeloproliterative Neoplasms; PMF, primary myelofibrosis; Retro, retrospective study; RIC-PBST, reduced-intensity conditioning peripheral blood stem cell transplantation; ICE, idarubicin, cytosinearabinoside, etoposide; FLAG, fludarabine, cytosine arabinoside, granulocyte colony-stimulating factor; CLARA, clofarabine, cytosine arabinoside; CB, cord blood; BMT, bone marrow transplantation; TBI, total body irradiation; flu, fludarabine; bu/Bu, busulfan; Mel, melphalan; Cy, cyclophosphamide; MAC, myeloablative conditioning; Col, clofarabine; Thio, thiotepa; FLAMSA, flu/cytarabine and amsacrine; MSD, matched sibling donor; URD, unrelated donors; MUD, matched unrelated; GvHD, graft-versus-host disease; TRM, Transplant-related mortality; NRM, nonrelapse mortality; NM, not mentioned.
