# Supplementary material for: Venetoclax‐Based Therapy for Early Relapse in Acute Myeloid Leukemia After Allogeneic Hematopoietic Stem Cell Transplantation: A Case Report and Minireview
Source: Cancer Rep (Hoboken). 2025 Dec 29;9(1):e70450. doi: 10.1002/cnr2.70450 (PMC12747801; doi:10.1002/cnr2.70450)
Supplement: Supplementary file 4 — Table S4: The studies investigating AZA ± DLI as a treatment of posttransplantation (the data were from PubMed between 2012 and 2022). [file CNR2-9-e70450-s005.docx]

TABLE S4. The studies investigating AZA ±DLI as a treatment of post-transplantation (The data were from PubMed between 2012 and 2022).

| study | Year | Diagnose:  numbers | Patients | Median age  (years) | Type of relapse | Median time to relapse  (months) | AZA  schedule | DLI | | | CR  (%) | ORR  (%) | Median Survival  (months) | 2-years OS  (%) | GVHD  (%) | TRM/  NRM  (%) |
| --- | --- | --- | --- | --- | --- | --- | --- | --- | --- | --- | --- | --- | --- | --- | --- | --- |
|  |  |  |  |  |  |  |  | NO. | Median  cycles | Total median CD3+ cell numbers/patient |  |  |  |  |  |  |
| Platzbecker et al.  (45) | 2012 | AML: 17  MDS: 3 | 20 | 58 (20-74) | CD34+ donor chimerism < 80% | 2.8 (1.9-97.7) | 75 mg/m^2^/d, d1-7 | Not combined | | | 50 | 50 | NM | 45 | 0 | NM |
| Oran et al.  (46) | 2020 | AML: 65  MDS: 22 | 87 | 57.5 (20-75) | Non-relapse | - | 32 mg/m^2^/d, d1-5  4(1-12) cycles | Not combined | | | - | - | 30.2 | NM | aGVHD: 25.5  cGVHD: 25.8 | NM |
| Webster et al.  (47) | 2021 | AML: 11  MDS: 5  t-MN:2 | 18 | 62 (19–72) | Non-relapse | - | 53.7 mg/m^2^/d, d1-5  8(1-12) cycles | Not combined | | | - | - | 29 | 57 | GVHD:5 | NM |
| Schroeder et al.  (51) | 2013 | AML: 8  MDS: 4  CMML: 1 | 13 | NM | Morph | 14.9 (0.6-56.3) | 100 mg/m^2^/d, d1-5;  75 mg/m^2^/d, d1-7;  6(4-8) cycles | 13 | 2 (1-4) | 5.0×10^6^/kg  (1.0-119.0×10^6^/kg) | 62 | 69 | NM | NM | aGVHD: 38  cGVHD: 46 | NM |
| Schroeder et al.  (48) | 2015 | AML: 124  MDS: 28  MPN: 2 | 154 | 55 (21-72) | Morph  Molec | 6.2 (0.6-111.6) | 100 mg/m^2^/d, d1-5;  75 mg/m^2^/d, d1-7;  4(4-14) cycles | 105 | 2 (1-7) | 31.2×10^6^/kg  (3.0-303.0×10^6^/kg) | 27 | 33 | NM | 29±4 | aGVHD: 34  cGVHD:20 | NM |
| Craddock et al.  (52) | 2016 | AML: 37 | 37 | 60 (40-71) | Non-relapse | - | 36 mg/m^2^/d, d1-5  No mentioned cycle | Not combined | | | - | - | NM | 49 (35-68) | aGVHD: 46  cGVHD: 27 | NRM:8 |
| Craddock et al.  (50) | 2016 | AML: 116  MDS: 65 | 181 | NM | Morph | 8.0 (1.0-71.0) | 75 mg/m^2^/d, d1-5;  No mentioned cycle | 69 | NM | NM | 15 | 25 | NM | 12.4 | NM | NM |
| Drozd-Sokołowska et al.  (53) | 2016 | AML: 6  MDS: 3 | 9 | 56 (15-78) | Morph | ≤6.0 | 75 mg/m^2^/d, d1-7;  3(1-6) cycles | 6 | 3 (1-7) | 40.0×10^6^/kg  (1.2-139.0×10^6^/kg) | 0 | 33 | 6.8 | 0 | NM | NM |
| El-Cheikh et al.  (54) | 2017 | AML: 13  MDS: 5 | 18 | NM | Non-relapse | - | 32 mg/m^2^/d, d1-5;  16(1-45) cycles | Not combined | | | - | - | NM | 72 | aGVHD: 11  cGVHD: 22 | 11 |
| Woo et al.  (55) | 2017 | AML: 26  MDS: 13 | 39 | 52 (23-76) | Morph  Molec | ≤3.3 | 75 mg/m^2^/d, d1-7;  3(1-6) cycles | Not combined | | | 8 | 31 | NM | 25 | aGVHD:74  cGVHD: - | NM |
| Karakulska-Prystupiuk et al.  (56) | 2018 | AML: 13  MDS: 6  CMML: 4  PMF: 1 | 24 | 60 (15-78) | Morph  Molec | ≤6.0 | 75 mg/m^2^/d, d1-7;  30 mg/m^2^/d, d1-7;  (1-9) cycles | 10 | 3 (1-7) | 2.0×10^6^/kg  (0.1-10.0×10^6^/kg) | Morph:  20  Molec:  43 | NM | Morph:  6.1 (0.7-13)  Molec:  21.2 (8.4-NR) | NM | aGVHD: 13  cGVHD: 18 | NM |
| Rautenberg et al.  (57) | 2020 | AML: 90  MDS: 49  MPN: 12 | 151 | 54 (19-71) | Morph  Molec | 4.9 (0.9-214.7) | 75 mg/m^2^/d, d1-7;  100 mg/m^2^/d, d1-5;  5(1-18) cycles | 105 | 2 (1-6) | 6.0×10^6^/kg  (0.5-116.0×10^6^/kg) | 41 | 46 | NM | 38±9 | NM | NM |
| Drozd-Sokołowska et al.  (58) | 2021 | AML: 16  MDS: 7 | 23 | 56 (21-66) | Morph | 3.5 (1.6- 93.5) | 75 mg/m^2^/d, d1-7;  100 mg/m^2^/d, d1-5;  32 mg/m^2^/d, d1-5;  37.5 mg/m^2^/d, d1-5;  3 (1-9) cycles | 11 | 2 (1-5) | 10.0×10^6^/kg  (1.0-116.0×10^6^/kg) | 13 | NM | 5.9 (3.4-13) | 8 (1-48) | aGVHD: 4  cGVHD: 0 | NM |
| Yoshimoto et al.  (59) | 2021 | AML: 38 | 38 | 61 (22-73) | Morph | 6.5 (0.6- 60.5) | 75 mg/m^2^/d, d1-7, or d1-5, or d1-3  32.5 mg/m^2^/d, d1-5;  No mentioned | 4 | NM | NM | 26 | 29 | NM | 26.1(13.4-40.6) | NM | NM |
| Liberatore et al.  (49) | 2022 | AML: 52  MDS: 19 | 71 | 56 (19-71) | Morph  Molec | 9.0 (1.0-112.0) | 75 mg/m^2^/d, d1-7;  32 mg/m^2^/d, d1-5;  4 (1-37) cycles | 33 | 2 (1-4) | 2.1×10^6^/kg  (0.1-62.5×10^6^/kg) | 38 | 49 | 7 .0 (1-90) | 41 (29-53) | aGVHD: 27  cGVHD: 18 | TRM:  1 (1-7) |
| Poiré et al.  (60) | 2022 | AML: 30  MDS: 19 | 49 | 60 (17-73) | Morph | 4.2 (2.6-16.8) | 100 mg/m^2^/d, d1-5;  35 mg/m^2^/d, d1-5;  3 (1-12) cycles | 12 | 1 (0-3) | NM | 20 | 22 | 6.0 (3.2-8.8) | NM | aGVHD: 5.1  cGVHD: 12.5 | NM |
| Wattebled et al.  (61) | 2022 | AML: 45  MDS: 18  CML: 2 | 65 | 58 (47-64) | Non-relapse | - | 32 mg/m^2^/day, d1-5  75 mg/m2/day, d1-5 | Not combined | | | - | - | NM | 56 (43.0–67.2) | NM | NM |

Abbreviation: AML, acute myeloid leukemia; MDS, myelodysplastic syndromes; CMML, chronic myelomonocytic leukemia; PMF, primary myelofibrosis; t-MN, therapy-related myeloid neoplasm; Morph, Morphological; Molec, Molecular; AZA, Azacytidine; DLI, Donor lymphocytes infusions; GvHD, graft-versus-host disease; NO., numbers; TRM/NRM, treatment-related mortality/non-relapse mortality; NM, Not mentioned; NR, Not reached.
