# Supplementary material for: Venetoclax‐Based Therapy for Early Relapse in Acute Myeloid Leukemia After Allogeneic Hematopoietic Stem Cell Transplantation: A Case Report and Minireview
Source: Cancer Rep (Hoboken). 2025 Dec 29;9(1):e70450. doi: 10.1002/cnr2.70450 (PMC12747801; doi:10.1002/cnr2.70450)
Supplement: Supplementary file 5 — Table S5: The studies of venetoclax‐based ± DLI therapy in AML and MDS relapse after transplantation (the data were from PubMed between 2012 and 2022). [file CNR2-9-e70450-s001.docx]

TABLE S5. The studies of Venetoclax-based ± DLI therapy in AML and MDS relapse after transplantation (The data were from Pubmed between 2012 and 2022).

| study | Year | Type of study | Diagnose (numbers) | Median age | Type of relapse | Median time to relapse  (months) | Venetoclax partner | Cycles of Venetoclax | DLI | | | CR  (%) | ORR  (%) | Median Survival  (months) | GVHD  (%) | TRM  (%) |
| --- | --- | --- | --- | --- | --- | --- | --- | --- | --- | --- | --- | --- | --- | --- | --- | --- |
|  |  |  |  |  |  |  |  |  | Numbers | Median  cycles | Median CD3+ cell numbers |  |  |  |  |  |
| Byrne et al.  (73) | 2020 | Retro | AML (16)  MDS (3)  CMML (1)  PMF (1) | 65 (35-74) | Morph | 5.7 (0.9-44.9) | AZA (52%)  DEC (24%)  LDAC (24%) | 3 (1-11) | NM | | | 33 | 57 | 7.8 (0.2-12.1) | NM | 43 |
| Amit et al.  (74) | 2021 | Retro | AML (22) | 65 (43-75) | Morph  Molec | 3.2 (1.0-6.0) | LDAC (4.5%)  HIDAC (9%)  AZA (23%)  Sorafenib (23%)  Gilteritinib (14%) | 2 (1-8) | 22 | 1 (1-3) | Sibling:  (5-10) × 10^6^/kg  Unrelated:  (1-5) × 10^6^/kg | 41 | 50 | 6.1 (0.73-11.4) | aGVHD: 23  cGVHD: 27 | NM |
| Joshi et al.  (70) | 2021 | Retro | AML (19)  MDS (10) | 58 (20–72) | Morph | 9.0(2.0-37.0) | DEC (62%)  AZA (28%)  LDAC (3.4%)  Gilteritinib (3.4%) | 2 (1-10) | Not combined | | | 28 | 38 | 2.6 (0.06-13.4) | aGVHD: 0  cGVHD: NM | NM |
| Schuler et al.  (71) | 2021 | Retro | AML (30)  MDS (2) | 54 (31-72) | Morph  Molec | 5.7 (1.1-67.8) | AZA (41%)  DEC (59%) | 2 (1-19) | 11 | 1 (1-3) | 5 (0.5–31) × 10^6^/kg | 33 | 47 | 3.7 (2.8-4.6) | NM | NM |
| Zucenka et al.  (75) | 2021 | Retro | AML (20) | 59 (20-71) | Morph | 7.2 (1.6-37.8) | LDAC+ Actinomycin D (100%) | 1-2 | 10 | 2 (1-11) | 25 (1–120) × 10^6^/kg | 70 | 75 | 13.1 | aGVHD: 10  cGVHD: NM | 0 |
| Bewersdorf et al.  (76) | 2021 | Retro | AML (32)  MDS (5) | 62 | Morph | NM | LDAC (11%)  AZA (73%)  DEC (16%) | NM | 8 | NM | NM | 13 | 32 | 4.7 (3.8-NR) | aGVHD: 43  cGVHD: 11 | NM |
| Zhao et al.  (77) | 2022 | Retro | AML (26) | 24-46 | Morph | 7.0 (3.2-18.4) | AZA (100%) | 6-8 | 26 | 6-8 | 29 (12–58) × 10^6^/kg | 26.9 | 61.5 | 9.5 (2.7-20.3) | Total GVHD: 23.1 | NM |
| Serpenti et al.  (78) | 2022 | Retro | AML (11) | 65 (31-72) | Morph  Molec | 6.5 (0.9-48.1) | AZA (91%)  DEC (9%) | NM | 5 | NM | NM | 36 | NM | NM | NM | NM |

Abbreviation: AML, acute myeloid leukemia; MDS, myelodysplastic syndromes; CMML, chronic myelomonocytic leukemia; PMF, primary myelofibrosis; Retro, retrospective study; Morph, morphological; Molec, molecular; AZA, azacytidine; DEC, decitabine; LDAC, low dose cytarabine; DLI, donor lymphocytes infusions; CR, complete remission; ORR, overall response rate; GvHD, graft-versus-host disease; TRM, Treatment-related mortality; NM, Not mentioned; NR, Not reached.
